# Supplementary material for: The Cause of Hereditary Hearing Loss in GJB2 Heterozygotes—A Comprehensive Study of the GJB2/DFNB1 Region
Source: Genes (Basel). 2021 May 1;12(5):684. doi: 10.3390/genes12050684 (PMC8147375; doi:10.3390/genes12050684)
Supplement: Supplementary file 1 [file genes-12-00684-s001.zip › Table S2.pdf]

Supplementary file_Table2_detected variants

patient	*GJB2(NM_004004.5)*	family history	gene	deafness type related to gene	genomic position	transcript	c.DNA position	protein position	zygosity	GnomAD Allele Frequency	ACMG class	ACMG class modified according to Oza et al.1	PubMed ID, rsnumber	Degree of hearing loss	Age at onset	additional findings
**part A**		**probably pathogenic variants detected in patients.**														
CZ-218517	c.35delG	compound heterozygote, variants detected in the trans position through analysis of the bam files	GJB2	DFNB1	Chr13(GRCh37):g.20763686del	NM_004004.5	c.35del	p.(Gly12Valfs*2)	0/1	**NFE 0.96%** (1209 /62320)	**Pathogenic** (PA2, PVS1, PS3, PS4, PM2, PM3, PP1, PP5, BS2, BS4, BP2)	**Pathogenic** (PVS1, PM3_VS, PS4, BA1)	9285800/ rs80338939	-	prelingual	
					Chr13(GRCh37):g.20763748A>G	NM_004004.5	c.-22-6T>C	p.?	0/1	**NFE 0.09%** (114/ 63209)	**VUS** (PM2, BP4)	**VUS** (PM2_S, BP4)	25401782/rs141962118			
CZ-9493	c.269T>C p.(L90P)	compound heterozygote, parents are heterozygous	GJB2	DFNB1	Chr13(GRCh37):g.20763452A>G	NM_004004.5	c.269T>C	p.(Leu90Pro)	0/1	**NFE 0.12%** (153/ 64380)	**Pathogenic** (PS3, PS4, PM1, PM2, PM3, PP1, PP3, PP5, BS4, BP2, BP6)	**Pathogenic**	10218527/rs80338945	mild/moderate	prelingual	
					Chr13(GRCh37):g.20763744T>G	NM_004004.5	c.-22-2A>C	p.?	0/1	**NFE 0.079%** (100/ 63234) **ASJ 0.45%** (47/ 5125**)**	**Likely pathogenic** (PS3, PM3, PP3)	**VUS** (BS1, PM3_S, PP1_M, PS3_P)	24039984/rs201895089			
SK-D715	c.-23+1G>A	sporadic	GPSM2	DFNB82 / Chudley-McCullough syndrome	Chr1(GRCh37):g.109444472_109444473delinsGT	NM_001321039.1	c.858_859delinsGT	p.(Tyr286*)	1/1	No frequency in any population	**Likely pathogenic** (PVS1, PM2)	**Likely pathogenic** (PVS1_S, PM2, PP4)	0/0	profound	2 years	hydrocephalus

CZ-1255717	c.35delG	compound heterozygote, parents are heterozygous	KARS1	DFNB89	Chr16(GRCh37):g.75669586A>C	NM_001130089.1	c.871T>G	p.(Phe291Val)	0/1	**NFE 0** **OTH 0.016%** (1/ 3065)	**VUS** (PM2, PP3)	**VUS** (PM2_S, PP3, PM3-S)	31116475/rs772410450	-	prelingual	
					Chr16(GRCh37):g.75668189A>G	NM_001130089.1	c.881T>C	p.(Ile294Thr)	0/1	**NFE 0.002%** (2/64564) **LAT 0.034%** (12/ 17706)	**VUS** (PP3, BS1)	**VUS** (PM2_S, PP3, PM3-S)	0/rs762673443			
SK-D618	c.269T>C p.(L90P)		LOXHD1	DFNB77	Chr18(GRCh37):g.44102213G>A	NM_144612.6	c.4936C>T	p.(Arg1646*)	0/1	**NFE 0.0065%** (4/ 30755)	**Pathogenic** (PVS1, PS4, PM2, PP1)	**Likely pathogenic** (PVS1_S, PM2)	0/rs960741408			
					Chr18(GRCh37):g.44114316C>A	NM_144612.6	c.4194G>T	p.(Arg1398Ser)	0/1	No frequency in any population	**VUS** (PM2, PP3)	**VUS** (PM2, PM3_S)	0/0			
SK-D914	c.35delG	sporadic	MPZL2	DFNB111	Chr11(GRCh37):g.118133799del	NM_005797.3	c.72del	p.(Ile24Metfs*22)	1/1	**NFE 0.12%** (159/ 64254) **ASJ 0.38%** (39/5130)	**Pathogenic** (PVS1, PS3, PM2)	**Likely pathogenic** (PVS1, PS3_M, PP1_S)	29982980/rs752672077	moderate	10 years	
SK-D194	c.35delG	sporadic	MYO15A	DFNB3	Chr17(GRCh37):g.18030477C>T	NM_016239.3	c.4030C>T	p.(Gln1344*)	0/1	No frequency in any population	**Likely pathogenic** (PVS1, PM2)	**Likely pathogenic** (PVS1_S, PM2)	0/0	profound	prelingual	
					Chr17(GRCh37):g.18045436G>A	NM_016239.3	c.5693G>A	p.(Arg1898Gln)	0/1	**NFE 0** **SAS 0.0033%** (1/ 15300)	**VUS** (PM2, PP3)	**VUS** (PM2)	0/rs756752580			
SK-D42	c.269T>C p.(L90P)	sporadic, male	POU3F4	DFNX2	ChrX(GRCh37):g.82763939_82763942del	NM_000307.4	c.607_610del	p.(Gln203Glufs*37)	1/1*hemizygous	No frequency in any population	**Pathogenic** (PVS1, PS4, PM2)	**Pathogenic** (PVS1_S, PS4, PM2)	7839145/rs876657719	profound	prelingual	
CZ-486412	c.35delG	-	SLC26A4	DFNB4 / PDS	Chr7(GRCh37):g.107302088T>C	NM_000441.1	c.2T>C	p.?	0/1	**NFE 0.015%** (14/ 45401)	**Pathogenic** (PVS1, PS4, PM2, PM3)	**Pathogenic** (PVS1_S, PS4, PM2, PM5_S)	14679580/rs111033302	-	prelingual	

					Chr7(GRCh37):g.107335095C>G	NM_000441.1	c.1371C>G	p.(Asn457Lys)	0/1	No frequency in any population	**Likely pathogenic** (PS1, PM1, PM2, PM5, PP3)	**Likely pathogenic** (PS1, PM1, PM2)	0/0			
CZ-115114	c.35delG	compound heterozygote, parents are heterozygous	SLITRK6	deafness and myopia	Chr13(GRCh37):g.86369404G>A	NM_032229.2	c.1240C>T	p.(Gln414*)	1/1	**NFE 0.0027%** (3/56278)	**Pathogenic** (PVS1, PS3, PP1, PP5)	**Pathogenic** (PVS1_S, PS3, PM2)	23543054/rs587777069		-	myopia
CZ-5209	c.109G>A p.(Val37Ile)	compound heterozygote, parents are heterozygous	STRC	DFNB16	del	NM_153700.2			0/1	-	**Pathogenic**	**Pathogenic**	11687802	mild/moderate	-	
					del	NM_153700.2			0/1	-	**Pathogenic**	**Pathogenic**	11687802			
**part B**		**VUS detected in patients**														
CZ-518309	c.35delG	-	CDH23	DFNB12/USH1D	Chr10(GRCh37):g.73434888G>T	NM_022124.5	c.1469G>T	p.(Gly490Val)	0/1	**NFE 0** **OTH 0.092%** (1/330)	**VUS** (PM2, PP3)		0/rs1227049	-	-	
					Chr10(GRCh37):g.73545419A>T	NM_022124.5	c.5744A>T	p.(Asp1915Val)	0/1	No frequency in any population	**VUS** (PM2, PP3)		0/0			
SK-D1275	c.35delG	sporadic	GATA3	Hypoparathyroidism, deafness and renal dysplasia	Chr10(GRCh37):g.8111526T>C	NM_001002295.2	c.1015T>C	p.(Cys339Arg)	0/1	No frequency in any population	**VUS** (PM1, PM2, PP3)		0/0	moderate	prelingual	3 years old patient and no hypoparathyroidism

																and renal dysplasia were reported.
CZ-176612	c.35delG	sporadic	KCNQ4	DFNA2	Chr1(GRCh37):g.41282965C>G	NM_005219.4	c.343C>G	p.(Leu115Val)	0/1	No frequency in any population	**VUS** (PM2, PP3)		0/0	moderate	10 years	
SK-D57	c.35delG	-	SALL1		Chr16(GRCh37):g.51171238C>T	NM_002968.2	c.3760G>A	p.(Val1254Ile)	0/1	**NFE 0.00090%** (1/56880) **LAT 0.0029%** (1/17293)	**VUS** (PM2, PP3, BP1)		0/rs755542004	profound	congenital	
CZ-9233	c.35delG	-	SLC22A4	DFNB60	Chr5(GRCh37):g.131657904G>A	NM_003059.2	c.680G>A	p.(Arg227His)	0/1	**NFE 0.057%** (73/64516) **SAS 0.078%** (24/15283)	**VUS** (PP3, BS1)		0/rs143140136	-	prelingual	
					Chr5(GRCh37):g.131676250C>T	NM_003059.2	c.1445-8C>T	p.?	0/1	**NFE 0.04%** (52/64492) **OTH 0.069%** (5/3608)	**Likely benign** (BS1, BP4)		0/rs372338512			

The origin of the patient (– CZ – Czech Republic, SK – Slovakia) is indicated before the patient´s identification. ACMG criteria and modified ACMG criteria for genetic hearing loss are shown.[19] Column PubMed ID show the first report of the detected variant.

Part A represent the patient reported as clarified for cause of hearing loss, part B represent the patients with variants classified as VUS, where the cause of hearing loss was probably not detected.

GnomAD allele frequency – the frequencies for European Non-Finnish (NFE) population the most related to Czech and Slovak patients are indicated. The popMax frequency, the maximum allele frequency detected in any of the population related in GnomAD is listed. Where the popMax frequency is the same as NFE, only NFE is indicated. The number of heterozygotes and all examined patients related to NFE and popMax frequency is indicated in the brackets.

No homozygotes for reported variants were detected in GnomAD except the Chr13(GRCh37):g.20763686del variant, where for NFE population only four homozygotes were reported.

ASJ – Ashkenazi Jewish

LAT - Latino

SAS – South Asian

OTH – Other population
